# Engaging Participants Through Hybrid Community-Centered Approaches: Lessons Learned During the COVID CommUNITY Public Health Research Program

**DOI:** 10.1177/15248399231221161

**Published:** 2024-01-05

**Authors:** Sujane Kandasamy, Riddhi Chabrotra, Zainab Khan, Dania Rana, Noor Suddle, Dipika Desai, Farah Khan, Rochelle Nocos, Scott A. Lear, Sonia S. Anand

**Affiliations:** 1 Department of Medicine, McMaster University, Hamilton, Ontario, Canada; 2 Department of Health Research, Methods, Evidence & Impact, McMaster University, Hamilton, Ontario, Canada; 3 Population Health Research Institute, McMaster University, Hamilton, Ontario, Canada; 4 Faculty of Health Sciences, Simon Fraser University, Burnaby, British Columbia, Canada

**Keywords:** health disparities, health research, Asian, minority health

## Abstract

Community-centered research studies can improve trust, cultural appropriateness, and accurate findings through meaningful, in-depth engagement with participants. During the COVID-19 pandemic, researchers shifted to implement pandemic-specific guidelines on top of already existing safety practices; these adjustments gave insight into bettering the structure of forthcoming research studies. At the Population Health Research Institute (PHRI)/McMaster University, the COVID CommUNITY study staff took field notes from their experience at the Ontario (ON) and British Columbia (BC) sites navigating an observational prospective cohort study during the pandemic. These field notes are outlined below to provide insight into culturally responsive, trust-centered, and communication-focused strategies used to improve hybrid research. A significant challenge the team overcame was obtaining blood sample collections by executing socially distanced sample collections outside of participants’ homes, coined “Porch Pickups.” Data collection was made more accessible through phone surveys and frequent virtual contact. To enhance recruitment strategies for sub-communities of the South Asian population, staff focused on cultural interests and “gift-exchange” incentives. Cultural awareness was prioritized through correct name pronunciation, conducting data collection in participant preferred languages, and using flexible approaches to data collection. These strategies were developed through weekly team meetings where improvement strategies were discussed, and concerns were addressed in real-time.

## Introduction

The COVID CommUNITY study, an interdisciplinary collaboration based at the Population Health Research Institution (PHRI)/McMaster University, was established in the winter of 2021 to understand seropositivity and risk factors for SARS-CoV-2 infection and vaccine confidence, interest, and access among diverse South Asian populations living in Ontario and British Columbia (Canada). More information about the eligibility criteria and preliminary findings can be found elsewhere (Anand et al., 2022). Furthermore, to simultaneously understand why there was a lack of trust, confidence, and interest in the vaccine (which have historical underpinnings) and access-related challenges and facilitators of community roll-out, we conducted a qualitative study across both provinces (Kandasamy et al., 2023). The COVID CommUNITY study was conducted in a hybrid format, with in-person participant recruitment and dry blood spot (DBS) collection and virtual survey completion and one-on-one interviews. We built partnerships with local vaccination centers (including soccer centers and banquet halls) and grassroots organizations involved in the vaccine roll-out to help identify and recruit participants. The depth and breadth of connections we built with partner organizations became the glue holding together this community-centered program of research. Furthermore, our research team consists of an interdisciplinary group of researchers and community partners, many of whom identify with South Asian ancestry (those who are originally from the Indian subcontinent). The term “South Asian” includes heterogeneity across country of origin, language, culture, and so on, so it is not possible or feasible to have full representation within a research team or among participants recruited into a research study. We recognize, acknowledge, and celebrate this vast diversity and instead focused on representation across the geographical catchment areas in which this study operates (including of the leadership team). This composition of our team contributed positively to our ability to build trust, strengthen community connections, and collaborate with local partners to achieve recruitment goals.

Once enrolled, participants completed the informed consent process and two visits (each including a DBS and set of surveys). Through the ebbs and flows of establishing, conducting, and producing various knowledge mobilization outputs, the research team garnered learnings on successful approaches to participant-centered data collection within the context of hybrid study designs. These approaches (along with examples) are shared below.

Visit the study website, describing our community engagement process, key deliverables, and co-designed knowledge mobilization products (e.g., participant toolkits, videos, and social media content) here: www.covidcommunity.mcmaster.ca

### Covid Adjustments

As the study was conducted through 2021–2023, our study team made COVID-19 adjustments in accordance with public health guidelines, which were mandated across stages (“Canadian COVID-19 Intervention Timeline,” 2023). To remain ahead of changing guidelines and to help all participants feel comfortable, our position was to plan proactively. These included wearing masks/shields, pausing in-person recruitment when mandated (i.e., during declared lockdowns), and regularly sanitizing during DBS collection. When recruiting, researchers were required to remain 6 ft apart from participants, and this made it difficult to convey important study details and collect important information. In a similar vein, it was difficult to continue collecting DBS samples during the lockdowns. To overcome some of these challenges and continue with timely data collection, we conducted brief, distanced, outdoor visits we referred to. as “Porch Pickups.” Essentially, “Porch Pick-ups” were outdoor home visits where team members collected bio-samples and completed any outstanding priority questions with enrolled participants.

The majority of data collection (via surveys) occurred using the REDCap software where all surveys were designed to be self-administered, sent via email, or completed over the phone by trained research staff. Due to the limited opportunities to meet in-person, the research team developed a condensed version of the health surveys (truncated versions with priority questions only) and prioritized phone completion. Building trust through voice-to-voice contact amid the shift to virtual data collection was an important and successful approach to limiting attrition.

### In-Person Engagement

Throughout this hybrid study, recruitment strategies were culturally-tailored to maximize engagement with South Asian communities living in both the Greater Toronto Hamilton Area and the Greater Vancouver Area. For example, when in-person activities were deemed permissible by public health authorities, it became even more of a priority to offer opportunities for community-building while prioritizing safety (i.e., employing proactive preventive measures). We conducted socially-distanced Bhangra workshops, Henna, bracelet-making, painting activities, and serving of culturally familiar favorites and specialties (e.g., single-serve and/or individually-packaged food items such as samosas). The selection and details of these events were decided through discussions and feedback from the study team and based upon past successful event attendance. Recruitment in both provinces was also conducted in partnership with faith-based organizations (e.g., Gurdwaras, Temples who emerged as key institutions during the pandemic waves as places to obtain pandemic information and in some cases, vaccine roll-out sites) to stay up-to-date with community priorities. These strategies centered changing community interests and were effective ways to engage, increasing recruitment and data collection opportunities. In addition, using a virtual gift-exchange approach to show gratitude for participant commitment/time investment prolonged participant engagement (a 25 dollars CAD gift card where participants could self-select from a diverse selection of grocery and general outlets) distributed after each completed study visit (two total). This dollar amount was decided by balancing funds available with previous feedback from other studies our research group has led in these catchment areas. Another participant-informed strategy to improve engagement and increase participant interest was to offer participants with aggregate study results, personalized COVID-19 antibody results, and Interheart risk scores (a validated measure of one’s future myocardial infarction risk based on current risk factors) (McGorrian et al., 2011). To increase engagement during recruitment events, colorful banners, posters, and easily identifiable red COVID CommUNITY T-shirts were used. For SA youth subcommunity recruitment, raffle draws, stickers, and snacks were also available. We measure success in recruitment and set goals for data completion by reviewing data weekly at team meetings. The goal was set at 80% data completion.

### Virtual Engagement

Employing virtual engagement strategies is important for the success of hybrid studies. We established a remote “calling team” composed of student volunteers and research assistants trained to engage with participants over the phone and/or Zoom to complete data collection in the form of short surveys (both in English and native preferences). Building a lasting virtual relationship with participants is crucial, as holding events for recruitment and follow-up was not always feasible during the pandemic. To develop strong participant-researcher relationships, we prioritized correct name pronunciations and offered different languages of communication. This allowed team members to build a cultural connection with the participants, establishing trust and comfort. To further build the relationship, a strategy that proved highly successful was relating to participant experiences throughout the pandemic (e.g., expressing resonance with the frustrations of not being able to visit with or hug family members outside of your home “bubble” during the early stages) . This encouraged conversation and developed trust in the researcher-participant relationship. Many questions in the study surveys involved discussing sensitive topics, such as mental health. It was important to understand these topics tend to be stigmatized in many South Asian communities. As such, it was recommended that research teams phrase these questions delicately to ensure participant comfort and to create nonjudgemental environments that normalize discussing mental health.

To reach participants virtually, various strategies were used, including using a number with or without caller ID. It was found that using numbers with a caller ID displayed, made the phone call more personable. While conducting the phone calls, finding a time when both the student caller and participant were available was difficult. The participants were called at various times on various days, including the morning, afternoon, and evening. It was found weekdays after 6 p.m. and weekends had the highest call-to-response ratio. Contact attempts were carefully documented on REDCap to provide context for others who contacted the same participants. Moreover, it was discovered that booking “Porch Pickups” and clinic visits immediately over the phone were advantageous over booking them later to avoid calling participants several times. Mass texts for booking porch pickups were also sent out to remind participants about the study. At the BC site location, Zoom calls were offered to provide virtual support (e.g., step- by-step guidance) to participants as they completed the DBS collection, a strategy that proved to be successful.

To supplement engagement opportunities for participants, we also conducted virtual panel discussions (virtual townhalls) featuring invited guests to speak on curated topics of interest and allowing time for open Q&A. We conducted these in partnership with physicians, public health professionals, faith leaders, and those with lived experiences to spark community interest, address knowledge gaps, and offer guidance in pandemic-related decision-making.

### Communication and Problem-Solving

Open communication and strong problem-solving skills within the research team were critical to helping meet study goals and optimize strategies (including regular dialogue between the virtual and on-the-ground teams). Throughout the study, weekly meetings were conducted with the student callers to discuss any difficulties that may have occurred during the phone calls. Some common inquiries included handling participant withdrawals, special requests, and contact detail changes. The team employed different collaborative strategies to maximize data collection. For example, phone scripts and a color-coded spreadsheet were created to navigate different scenarios and inquiries students faced. Similarly, students organized their calling shifts in a spreadsheet to communicate their availabilities. This ensured that calling was dispersed throughout the day and week to reach participants at their convenience. Finally, to increase data completeness among all groups of participants, students split into three teams to contact first-priority (participants who have outstanding visit one and visit two surveys), second-priority (participants who have minimal outstanding surveys in either visit), and neighborhood-based groups (participants within a designated geographical area with outstanding DBS data).

## Conclusion

It is evident that research communities will engage more closely with hybrid designs after the effects of COVID-19. Considering careful methodology when carrying out research for hybrid studies has proven valuable, especially due to the increasing vulnerability of several populations during the pandemic, including in South Asian communities (Thobani & Butt, 2022). Many adjustments were required when conducting the hybrid study and it is vital to develop effective strategies to minimize these difficulties. Some recommendations include establishing trust and building a connection with participants to maintain a virtual relationship, developing responsive and proactive study recruitment approaches, and working within public health guidelines to create opportunities for engaging community connections.

**Figure 1.**
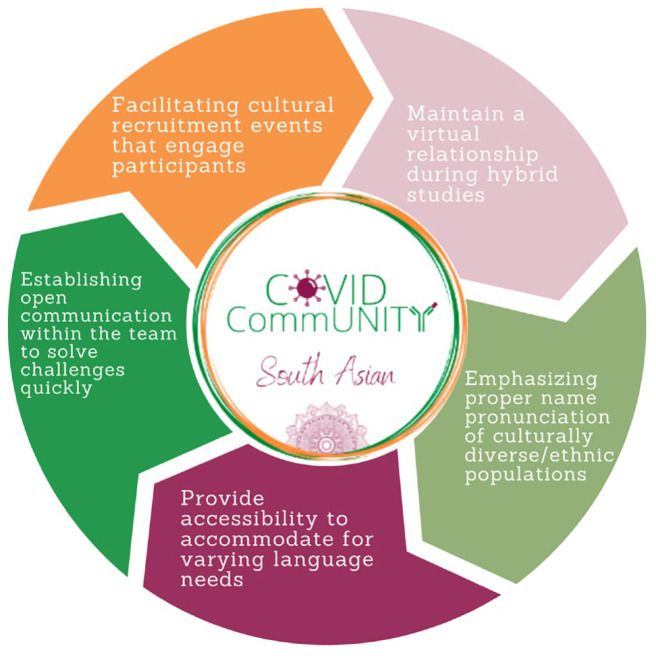
Recommendations for conducting an effective hybrid study (based on key learnings from the COVID CommUNITY study).
